# Four-flanged polypropylene optic piercing technique for scleral fixation of multifocal intraocular lens

**DOI:** 10.1186/s12886-023-03133-7

**Published:** 2023-09-26

**Authors:** Youngsub Eom, Eunheh Koh, Seul Ki Yang, Soo Kim, Sungtae Yi, Hyun Sun Jeon, Seong-Jae Kim, Jason So, Jong Suk Song, David L Cooke

**Affiliations:** 1 Department of Ophthalmology, Korea University Ansan Hospital, Gyeonggi-do, Republic of Korea; 2 Department of Ophthalmology, Korea University College of Medicine, Seoul, Republic of Korea; 3 Department of Ophthalmology, Emory University School of Medicine, Atlanta, GA USA; 4 Medical College of Georgia, Augusta University, Augusta, GA USA; 5 Space Optics Laboratory, Department of Astronomy, Yonsei University, Seoul, Republic of Korea; 6 Satellite system 2 Team, Hanwha Systems Co., Ltd, Gyeonggi-do, Republic of Korea; 7 BNeye Clinic, Seoul, Republic of Korea; 8 Department of Ophthalmology, Seoul National University College of Medicine, Seoul National University Bundang Hospital, Seongnam, Republic of Korea; 9 Department of Ophthalmology, Gyeongsang National University Hospital, Gyeongsang National University College of Medicine, Jinju, Republic of Korea; 10 Institute of Health Science, Gyeongsang National University, Jinju, Republic of Korea; 11 Great Lakes Eye Care, Saint Joseph, MI USA; 12 Department of Neurology and Ophthalmology, College of Osteopathic Medicine, Michigan State University, East Lansing, MI USA

**Keywords:** Flanged, Scleral fixation, Optic piercing

## Abstract

**Background:**

To evaluate the feasibility of creating flanges using an optic piercing technique with a 6 − 0 polypropylene monofilament for scleral fixation of dislocated one-piece diffractive multifocal intraocular lenses (IOLs).

**Study Design:**

Experimental study and case series.

**Subjects:**

Optical bench test and eyes with IOL dislocation.

**Methods:**

Two separate 6 − 0 polypropylenes were penetrated twice at the opposite peripheral optic of the TECNIS Synergy IOL (Johnson & Johnson Vision). The root mean square of the modulation transfer function (MTF_RMS_), at between + 1.00 and − 4.00 D of defocus, was measured in the TECNIS Synergy IOL both with and without optic piercing in the optical bench study. This case series included three eyes from two patients who underwent scleral-fixation of multifocal IOLs using the four-flanged polypropylene optic piercing technique. The postoperative corrected distance visual acuity (CDVA) at 4 m, the uncorrected near visual acuity (UNVA) at 40 cm, and IOL centration were evaluated.

**Results:**

The optical bench test showed no differences in MTF_RMS_ values measured in the TECNIS Synergy IOL, either with or without optic piercing at all defocuses. In all three case series, the postoperative CDVA at 4 m was 20/20 and UNVA at 40 cm was J1. Postoperative anterior segment photographs showed good centration of IOLs in all cases.

**Conclusion:**

The four-flanged polypropylene optic piercing technique for multifocal IOL scleral fixation can provide excellent clinical outcomes and IOL stability after surgery without diminishing the performance of the multifocal IOLs.

## Background

Multifocal intraocular lenses (IOLs) can provide excellent vision at both distance and near and/or intermediate distances [1–3]. However, accurate IOL positioning with no tilt or decentration is necessary for accurate postoperative refraction and patient satisfaction [4, 5]. Therefore, in order to preserve good visual outcome, refixation of a dislocated multifocal IOL requires a surgical technique that allows proper positioning of the IOL with respect to the type and design of the dislocated multifocal IOL [6].

A flanged intrascleral haptic fixation technique developed by Yamane et al., and a double-flanged intrascleral fixation technique using 5 − 0 or 6 − 0 polypropylene proposed by Canabrava et al. are both pioneering surgical techniques that can reveal new horizons for IOL scleral fixation [7–9]. The flanged intrascleral haptic fixation technique has the advantage of short surgical time and good long-term postoperative stability [10, 11]. If the three-piece IOL is dislocated, the dislocated IOL can be reused to perform intrascleral haptic fixation [6, 12]. However, the double-flanged intrascleral haptic fixation technique requires a three-piece IOL and cannot be used for fixation of one-piece IOLs. By contrast, the Canabrava double-flanged intrascleral fixation technique using polypropylene can be easily applied to scleral fixate one-piece IOLs, IOLs with eyelets, or loop haptic IOLs [13, 14].

When the Canabrava double-flanged technique is used to scleral fixate multifocal IOLs with a C-loop haptic, a method of tying polypropylene to the optic-haptic junction, such as the cable tie technique, can be used [15]. Although the Canabrava technique can provide excellent IOL stability after scleral fixation, it is complex and technically difficult. The piercing technique, which is one method of fixing 5 − 0 or 6 − 0 polypropylene to the IOLs with C-loop haptics, can be applied to the haptic or optic [16]. In this method, after inserting a needle into a haptic or optic, a polypropylene thread is inserted into the lumen of the needle, and then the needle is pulled out to fix the thread. However, inserting the needle into the IOL haptics risks dislocation of the IOL by cheese-wiring, and piercing the polypropylene in the optics may distort the IOL optic. This study aimed to evaluate the feasibility of creating the flange using the optic piercing technique with a 6 − 0 polypropylene monofilament for scleral fixation of multifocal IOLs.

## Materials and methods

### Experimental study

#### Multifocal IOL optic piercing using 6 − 0 polypropylene

**Fig. 1:**
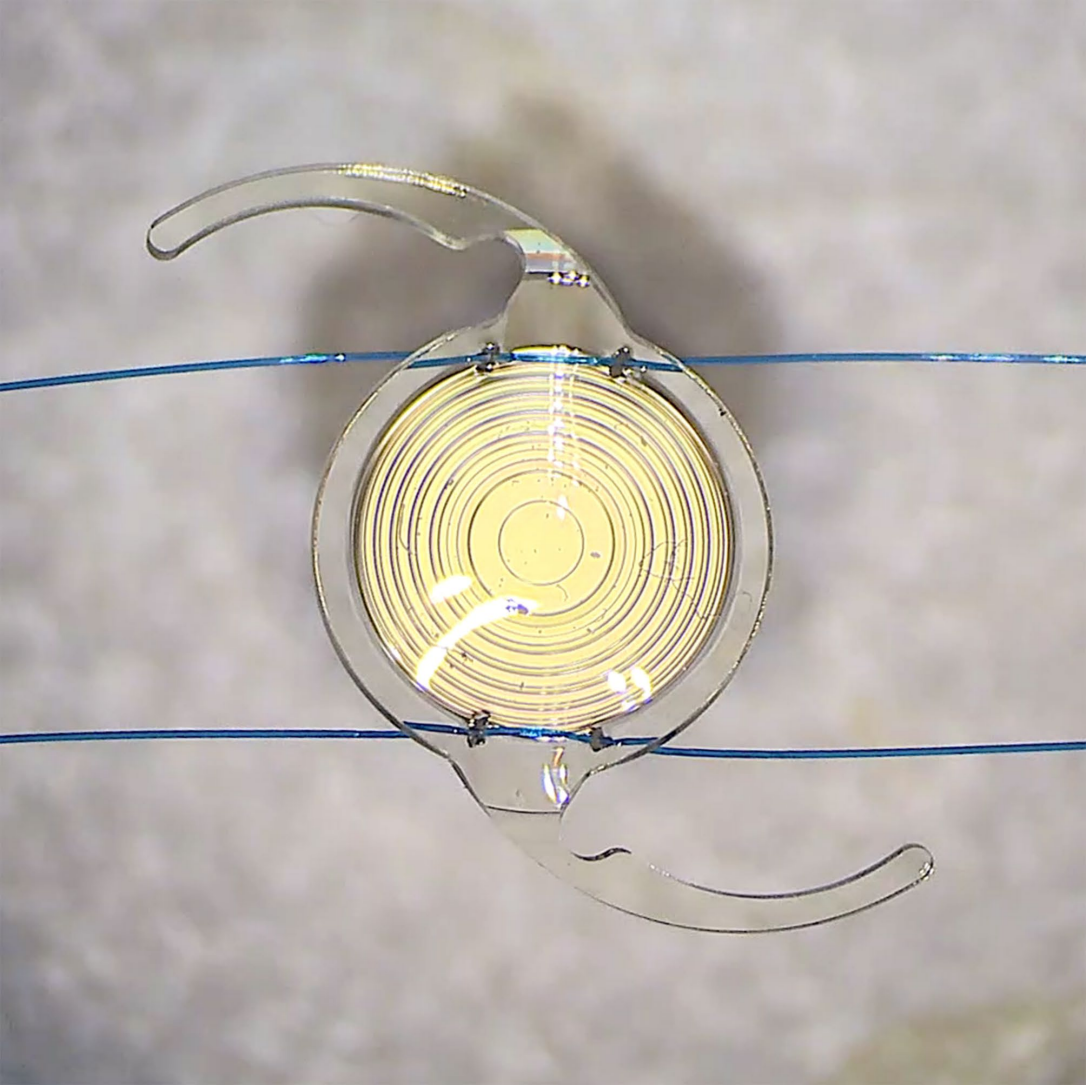
The preparation of the TECNIS Synergy IOL (Johnson & Johnson Vision) with optic piercing for the optical bench study using two separate 6 − 0 polypropylene monofilaments

Two TECNIS Synergy IOLs (Johnson & Johnson Vision) with 20 D were used in the experimental study (Table 1). A 30 G needle (Jung Rim Medical Industrial Co. Ltd., Seoul, Republic of Korea) was used to pierce the periphery of the IOL optic near the optic-haptic junction. Then, a 6 − 0 polypropylene monofilament (Ailee Co. Ltd, Busan, Republic of Korea) was docked into the lumen of the 30 G needle. Then the needle was withdrawn to allow the 6 − 0 polypropylene to pierce the IOL optic. The 6 − 0 polypropylenes in the region that passes through the IOL optic were pinched and flattened with a needle holder. This method was repeated for the adjacent peripheral optic, allowing the 6 − 0 polypropylene to penetrate the IOL optic twice near one haptic. A separate segment of 6 − 0 polypropylene monofilament was used to repeat the piercing process in the peripheral optic adjacent to the opposite haptic (Fig. 1).

**Table 1:** Characteristics of the IOLs used in this study

Parameter	TECNIS Synergy	AcrySof IQ PanOptix
Material	Soft, foldable hydrophobic acrylic with UV light-absorbing material and violet light-filtering chromophore	Hydrophobic Acrylate/Methacrylate Copolymer with UV-light and blue-light filter
Refractive index	1.47	1.55
Abbe number	55	37
Overall length, mm	13.0	13.0
Optic size, mm	6.0	6.0
Optic design	One-piece, biconvex, trifocal Wavefront-designed anterior aspheric surface and ChromAlign technology Posterior diffractive surface (15 rings)	One-piece, biconvex, trifocal Anterior diffractive surface on 4.5 mm portion of the optic zone with + 2.17 D and + 3.25 D add power at the IOL plane
Haptic design	Tri-Fix haptics offset from optic; C-haptic	Modified L-haptic

IOLs = intraocular lenses; UV = ultraviolet.

### Optical bench system

An optical bench system with the same configuration mentioned in the previous study was also used in this study [17]. A 555 nm LED light was used to illuminate the 1951 United States Air Force (1951 USAF) resolution test chart, which was placed so that element 3 of group 2 of the 1951 USAF resolution test chart would be 15 cycles per degree (CPD), which is approximately equal to 20/40 visual acuity. A pupil camera was used to determine the IOL centration. The model eye consisted of an aberration-free artificial cornea and an artificial anterior chamber made of N-BK7 (DG100 × 100-600). The anterior chamber was filled with a balanced salt solution [17, 18].The IOL was fixed at the lens adapter, which was mounted on an XYZ translation and positioned in the artificial anterior chamber. Then the center of the lenses, the IOL, and the complementary metal-oxide-semiconductor (CMOS) camera (BFS-U3-120S4M-CS; FLIR Systems Inc., Wilsonville, OR) were aligned. The trial lens was placed in front of the artificial cornea to obtain a defocus image of between − 4.00 D and + 1.00 D at 0.50 D intervals [17, 19]. At each defocus, the image formed by the TECNIS Synergy IOL with and without the optic piercing using 6 − 0 polypropylene was captured by the CMOS camera. The horizontal and vertical directions in element 3 of group 2 of the obtained image of the 1951 USAF resolution test chart were converted to through-focus modulation transfer function (MTF) using MatLab (Mathworks, Inc., Natick, MA) [17]. The MTF measurement with a 5.0 mm pupil size was performed four times, and the average value was obtained. The root mean square of the horizontal and vertical MTF (MTF_RMS_) was compared between the TECNIS Synergy IOL both with and without optic piercing using 6 − 0 polypropylene [17, 19].

## Clinical case series

The Ethics Committee of Korea University Ansan Hospital, Gyeonggi-do, Republic of Korea approved this retrospective case series study (no. 2022AS0225). All research and data-collection protocols complied with the tenets of the latest Declaration of Helsinki.

### Study population

This study was a retrospective review of the medical records of patients who underwent multifocal IOL scleral fixation using the 6 − 0 polypropylene optic piercing technique due to the subluxation or dislocation of multifocal IOL at the Korea University Ansan Hospital between 1 July and 30 July 2022.

### Surgical technique

**Fig. 2:**
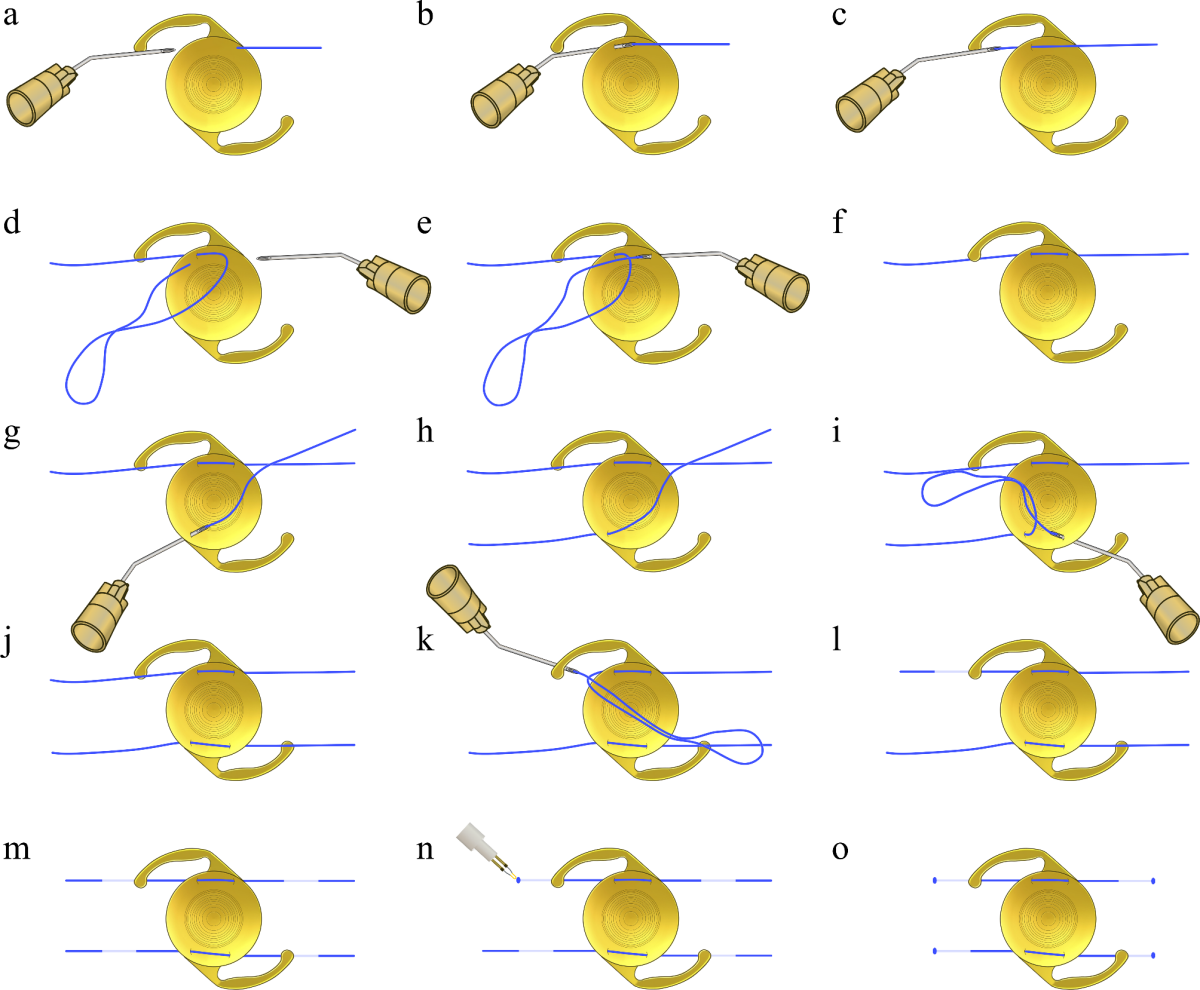
The four-flanged polypropylene optic piercing technique for scleral fixation of a one-piece multifocal intraocular lens (IOL) involves optic piercing using two separate 6–0 polypropylene sutures. After inserting the 30G needle into the peripheral optic of the IOL, the needle is withdrawn by docking a 6 − 0 polypropylene monofilament suture into the needle lumen so that the 6 − 0 polypropylene suture is pierced through the optic (**a** and **b**). The other end of the 6 − 0 polypropylene suture is then pierced through the adjacent peripheral optic of the IOL (**c** and **d**). Similarly, another 6–0 polypropylene monofilament suture is pierced twice into the opposing peripheral optic of the IOL (**e**). Four ends of the 6–0 polypropylene sutures are externalized to 2.5 mm posterior to the limbus using a 30G needle. Flanges are made at four ends of the 6–0 polypropylene monofilament sutures by heating with a cautery (**f**)

Four corneal paracentesis wounds were made at the 2, 4, 8, and 10 o’clock positions. The 6 − 0 polypropylene was inserted into the anterior chamber through the side port at the 8 o’clock position, and a 30G needle was inserted into the anterior chamber through the side port at the 4 o’clock position (Fig. 2a). The 30G needle pierced the peripheral optic of the IOL while applying counterforce from the back using forceps, and the 6 − 0 polypropylene was docked into the lumen of the needle (Fig. 2b). The needle was then pulled out so that the polypropylene passed through the IOL optic and out of the eye (Fig. 2c). The area of the 6 − 0 polypropylene suture that will pass through the IOL optic was flattened by pinching it with a needle holder from the outside of the eye. Then, the opposite end of the 6 − 0 polypropylene suture was inserted into the anterior chamber from the side port at the 8 o’clock to the 2 o’clock position and externalized. The end of the 6–0 polypropylene was reinserted into the anterior chamber through the side port at the 2 o’clock position and a 30G needle was inserted into the anterior chamber through the side port at 8 o’clock (Fig. 2d). A 30G needle was then used to pierce the adjacent peripheral optic of the IOL while counterforce was applied from the back using forceps (Fig. 2e). The 6 − 0 polypropylene was docked into the lumen of the needle before the needle was pulled out so that the polypropylene passed through the IOL optic and out of the eye (Fig. 2f). Similarly, the opposite peripheral optic of the IOL was pierced twice using another 6 − 0 polypropylene monofilament through the side port at the 2 and 10 o’clock positions (Fig. 2g and j). Next, one end of the monofilament was moved from the side port from the 4 o’clock to the 10 o’clock position and reinserted into the anterior chamber through the side port at 10 o’clock. Then, a 30G needle was inserted 2.5 mm posterior to the limbus at the 4 o’clock position (Fig. 2k). The end of the 6 − 0 polypropylene was docked in the lumen of the 30G needle, and the end of the polypropylene was externalized through the needle (Fig. 2l). The opposite end of the polypropylene monofilament was externalized 2.5 mm posterior to the limbus through a 30G needle at the 8 o’clock position. Similarly, both ends of a separate 6 − 0 polypropylene monofilament were externalized 2.5 mm posterior to the limbus at the 2 and 10 o’clock positions respectively (Fig. 2m). While checking the IOL centration, flanges were made at the four ends of the 6 − 0 polypropylene monofilaments using a high-temp cautery (Fig. 2n and o).

### Patient evaluation

The postoperative uncorrected distance visual acuity (UDVA), the corrected distance visual acuity (CDVA) at 4 m, and the uncorrected near visual acuity (UNVA) at 40 cm were measured four weeks after surgery, and the residual spheres, cylinders, and sphere equivalents identified by manifest refraction were recorded. Postoperative ocular, corneal, and internal total high-order aberration (HOA) were measured using a wavefront analyzer (KR-1 W; Topcon, Tokyo, Japan). IOL centration was assessed using anterior segment photographs. The postoperative patient perception of photic phenomena (e.g., glare, starbursts, and halos) was evaluated using a questionnaire, with accompanying illustrations [20, 21]. The incidence of photic phenomena was graded from 1 to 5 (the higher the score, the more frequent the phenomena: grade 1, never; grade 2, rarely; grade 3, sometimes; grade 4, often; and grade 5, always). The degree of discomfort was graded from 1 to 5 (the higher the score, the more severe the discomfort: grade 1, not at all; grade 2, very little; grade 3, somewhat; grade 4, quite a lot; and grade 5, very uncomfortable) [20, 21]. Additionally, any intraoperative and postoperative complications were recorded.

### Statistical analyses

Descriptive statistics for all experimental data were obtained using statistical software (version 21.0; IBM Corp., Armonk, NY, USA). A student *t*-test was conducted to compare the MTF_RMS_ between the TECNIS Synergy IOL with and without optic piercing using 6 − 0 polypropylene. P < 0.05 was considered statistically significant.

## Results

### Optical bench performance

**Fig. 3:**
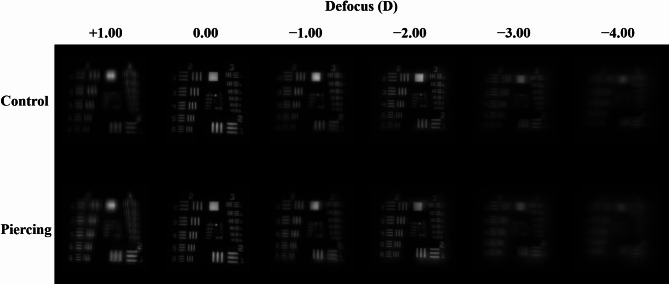
Representative captured images of the 1951 United States Air Force resolution test chart using the TECNIS Synergy IOL with and without optic piercing using 6 − 0 polypropylene. The minus (–) diopter defocus represents near distance

**Fig. 4:**
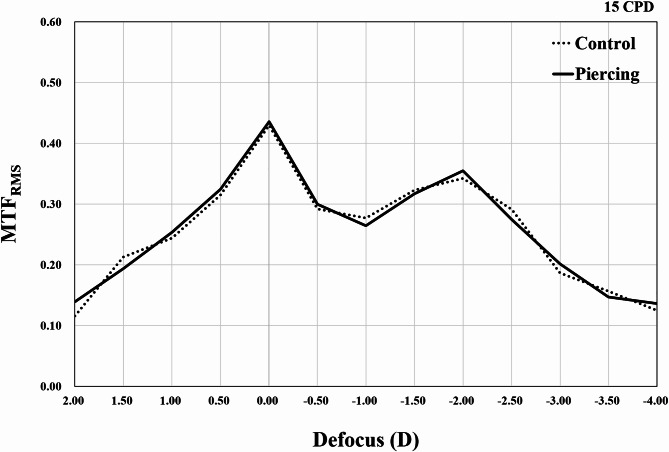
A comparison of the root mean square of modulation transfer function (MTF) values for a pupil size of 5.0 mm in the horizontal and vertical directions (MTF_RMS_) between TECNIS Synergy IOLs with (piercing; solid line) and without (control; dotted line) optic piercing using 6 − 0 polypropylene sutures

Figure 3 shows representative captured images of the 1951 USAF resolution test chart from the TECNIS Synergy IOL with and without optic piercing using 6 − 0 polypropylene. For both cases, the image was easily identifiable until − 3.00 D was added. Additionally, when − 4.00 D was added, the image was similarly blurred in both cases. MTF analysis showed that the TECNIS Synergy IOL with and without optic piercing using 6 − 0 polypropylene showed similar MTF_RMS_ graphs. There were no significant differences in MTF_RMS_ values between the TECNIS Synergy IOL with and without optic piercing using 6 − 0 polypropylene at all defocusings (Fig. 4). The highest MTF_RMS_ values (0.435 and 0.429, respectively) were obtained at 0.00 D of defocus and the second peak was obtained at a defocusing of − 2.00 D of defocus (0.355 and 0.342, respectively) in both the TECNIS Synergy IOL with and without optic piercing.

### Case presentations

#### Case 1

**Fig. 5:**
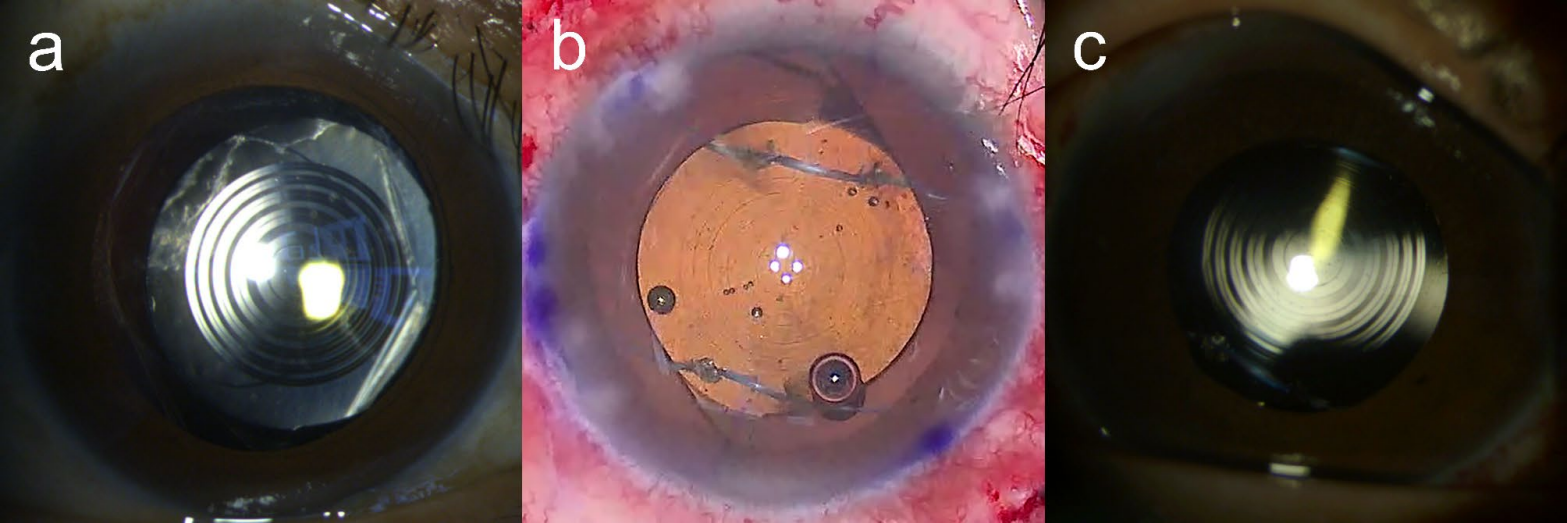
Preoperative anterior segment photograph (**a**), intraoperative photograph after surgery (**b**), and postoperative month one anterior segment photograph (**c**) of the right eye of case 1 using a PanOptix toric intraocular lens

A 51-year-old man with one year history of phacoemulsification with PanOptix toric IOL placement in both eyes (AcrySof IQ® PanOptix; Alcon Laboratories, Inc., Fort Worth, TX, USA) presented with an IOL subluxation in his right eye. Visual acuity in his right eye had decreased due to IOL subluxation following Nd:YAG laser capsulotomy at another hospital one month previously. Slit-lamp examination showed an IOL subluxation through the large capsulotomy site in the inferonasal direction (Fig. 5a). The patient wanted to restore both near and far vision and agreed to rescue a subluxated multifocal toric IOL through scleral fixation of the existing IOL. He underwent a scleral fixation 2.5 mm posterior to the limbus via the four-flanged polypropylene optic piercing technique with polypropylene flanges oriented to properly align the toric IOLs. One month after surgery, the patient’s UDVA was 20/25, CDVA was 20/20 with a refractive error of + 0.25 − 0.75 × 90°, and UNVA at 40 cm was J1 in his right eye. The postoperative ocular, corneal, and internal HOA in the 4-mm optical zone were 0.193 μm, 0.096 μm, and 0.165 μm, respectively. The IOL showed good centration both in the intraoperative photograph at the end of the surgery and the postoperative slit-lamp examination (Fig. 5b and c). The patient reported no glare, starbursts, or distortion (frequency grade 1), except for halos, which are sometimes experienced (frequency grade 3) but don’t cause any inconvenience (severity grade 1).

### Cases 2 and 3

A 58-year-old man with a five-year history of bilateral cataract surgery with placement of PanOptix and PanOptix toric IOLs was referred for reduced visual acuity caused by IOL subluxation in his right eye and subluxation of scleral fixated multifocal IOL in his left eye. His past ocular history included bilateral pars plana vitrectomy one month after the cataract surgeries and Nd:YAG posterior capsulotomy.

**Fig. 6:**
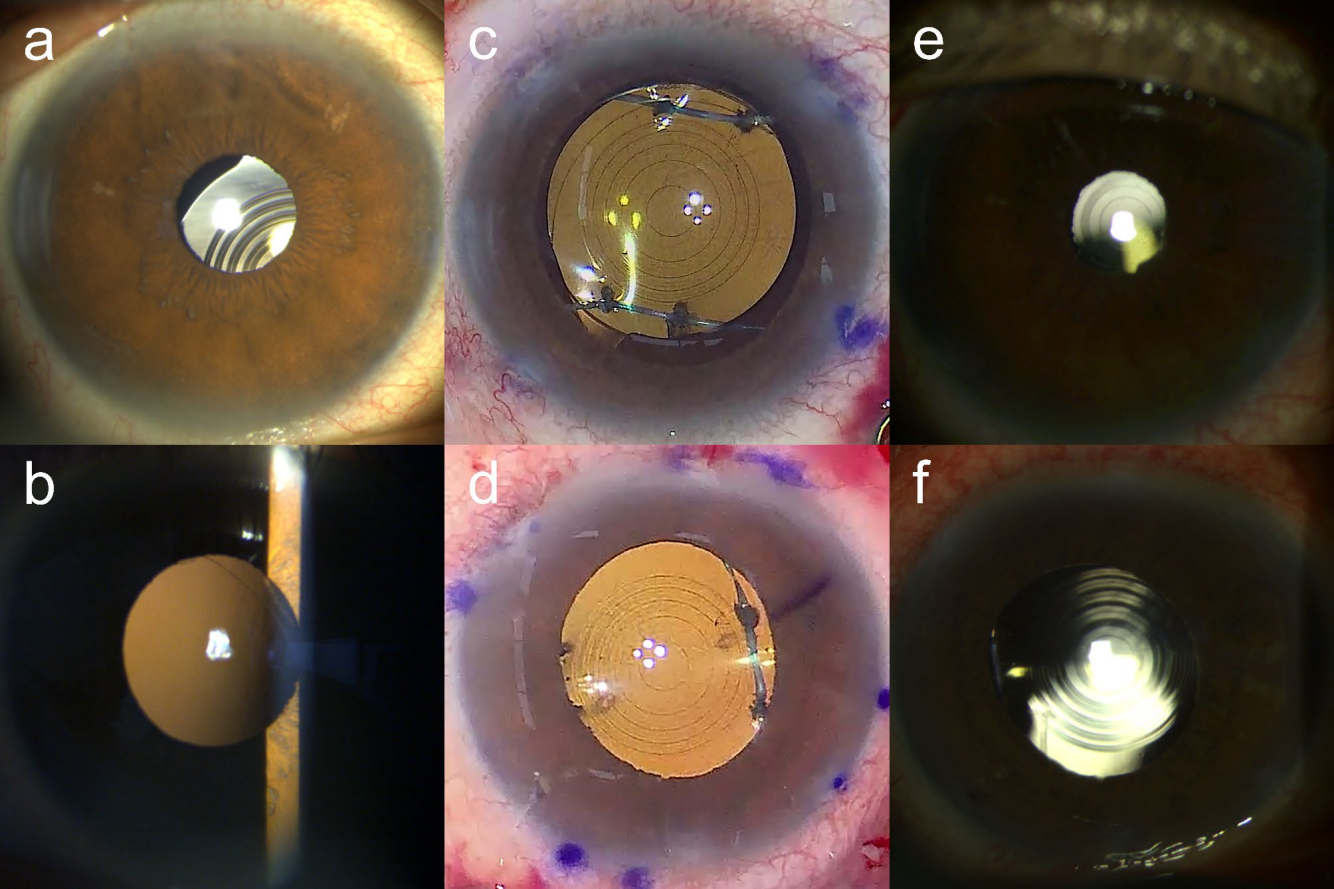
Preoperative anterior segment photographs (**a** and **b**), intraoperative photographs after surgery (**c** and **d**), and postoperative week three and four anterior segment photographs (**e** and **f**) of the right (**a**, **c**, and **e**) and left (**b**, **d**, and **f**) eyes of cases 2 and 3 using PanOptix and PanOptix toric intraocular lenses

A slit-lamp examination showed subluxed IOL in the right eye and posterior dislocation of the IOL optic secondary to ruptured optic-haptic junction in his left eye (Fig. 6a and b). He underwent scleral fixation of the IOLs in both his eyes 2.5 mm posterior to the limbus using the four-flanged polypropylene optic piercing technique. Three to four weeks after surgery, the patient’s UDVA was 20/20 in the right eye and 20/25 in the left eye, CDVA was 20/20 in both eyes with a refractive error of − 0.25 − 0.50 × 10° in the right eye and + 0.25 − 0.50 × 170° D in the left eye, and UNVA at 40 cm was J1 in both eyes. The postoperative ocular, corneal, and internal total HOA in the 4-mm optical zone were 0.316 μm, 0.194 μm, and 0.175 μm, respectively, in the right eye, and were 0.219 μm, 0.160 μm, and 0.272 μm, respectively, in the left eye. In both eyes, the IOL showed good centration both in the intraoperative photograph at the end of the surgery and the postoperative slit-lamp examination (Fig. 6c and f). The patient reported no glare, starbursts, halos, or distortion (frequency grade 1).

## Discussion

This study investigated the feasibility of performing a scleral fixation of a dislocated one-piece diffractive multifocal IOLs using the optic piercing technique with 6 − 0 polypropylene monofilaments to create flanges. The results of the optical bench test demonstrated that the optical performance of the multifocal IOL, whereby the IOL optic was pierced twice with two separate 6 − 0 polypropylenes in the periphery of the optic, was similar to that of the multifocal IOL without optic piercing. In addition, the clinical case series showed that three eyes of two patients who underwent scleral fixation via piercing the diffractive multifocal IOL optic with 6 − 0 polypropylene showed good postoperative clinical outcomes.

**Fig. 7:**
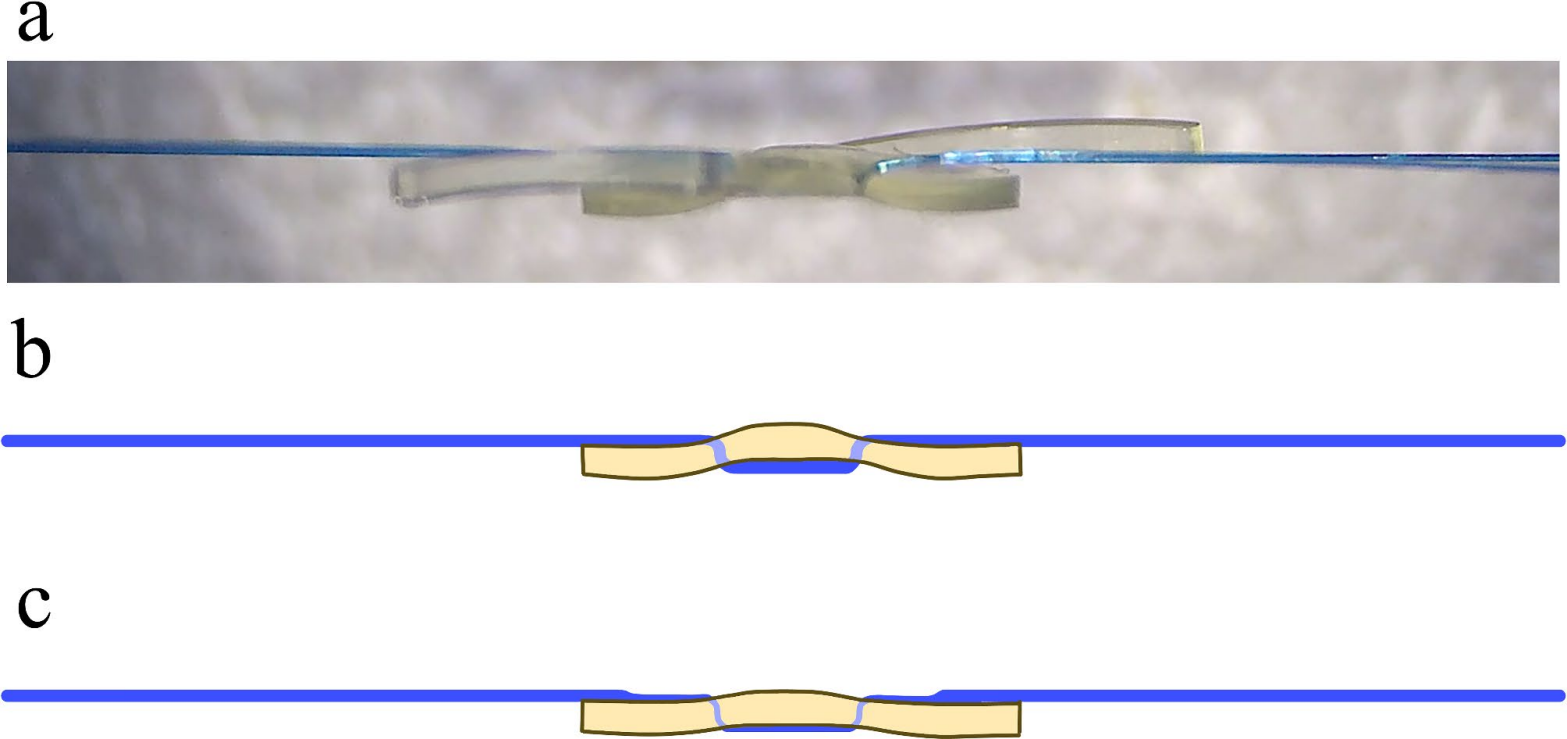
Intraocular lens (IOL) optic distortion caused by 6 − 0 polypropylene (**a**). In order to reduce distortion caused by the thickness of the 6 − 0 polypropylene (**b**), the area passing through the optic was flattened with forceps (**c**)

In a multifocal IOL with a specialized optic such as a diffractive ring, any defects or damage to the optic can cause severe clinical discomfort and reduced visual acuity [22]. When piercing the IOL optic with 6 − 0 polypropylene, the relatively thick 6 − 0 polypropylene can distort the IOL optic, which is what was observed during the preparation of the IOL for the optical bench test (Fig. 7a). If optic distortion occurs near the center of the optic, the function of the multifocal IOL will be greatly reduced. The thicker the polypropylene passing through the IOL optic, the greater the distortion of the IOL optic caused by the volume effect (Fig. 7b). Therefore, the 6 − 0 polypropylene suture was flattened with a needle holder to reduce distortion before passing through the optic (Fig. 7c). In the case series, no optical distortion was observed in the anterior segment photographs after surgery, and no photic phenomena other than halos were reported.

IOL scleral fixation using 9 − 0 or 10 − 0 polypropylene has been performed using various methods [6, 23]. Although it was first introduced as a trans-scleral fixation using 10 − 0 polypropylene, 10 − 0 polypropylene has been shown to degrade over time [23–25]. In addition, if a thin thread is tied to the optic-haptic junction of a one-piece IOL made of soft material, the optic-haptic junction could be cut due to the tension of the thread, as seen in case 3. Therefore, to obtain long-term stability, the scleral fixation was performed with thicker threads [26, 27]. The four-flanged intrascleral fixation technique using 5 − 0 or 6 − 0 polypropylene can be easily applied to the scleral fixation of one-piece IOL [13, 14] Hwang et al. reported encouraging clinical results after applying the four-flanged intrascleral fixation technique using 5 − 0 polypropylene to an extended depth of focus toric IOL and a bifocal hydrophobic IOL with loop haptics [13]. The four-flanged intrascleral fixation technique has the advantages of short surgery time and excellent postoperative stability but can only be applied to IOLs with loop haptics [13]. Because many multifocal IOLs have C-loop haptics [16], the optic-piercing technique was devised to allow application of the four-flanged intrascleral fixation technique to C-looped or double C-looped haptic IOLs.

Domingues et al. first reported the optic piercing technique for IOL scleral fixation using 10 − 0 polypropylene sutures, known as ‘cupid fixation’. In this technique, a double-armed 10 − 0 polypropylene suture was passed through the optic once, with one thread passing under the optic and the other thread passing over the optic. Then, both threads with needles are passed through the sclera for fixation [28]. The optic piercing technique using 6 − 0 polypropylene sutures for scleral fixation was introduced by Assia et al. They used 6 − 0 polypropylene sutures and performed scleral fixation by creating a flange [29, 30]. These studies demonstrated the safety and effectiveness of the optic piercing technique for monofocal IOL scleral fixation [28–30]. In this study, the optic piercing technique, in which the IOL optic was pierced twice with two separate 6 − 0 polypropylene sutures, was applied to subluxated diffractive multifocal IOLs. To the best of our knowledge, this study is the first to report the use of the optic piercing technique with 6 − 0 polypropylene sutures for subluxated multifocal IOLs. The results of this study confirm that the four-flanged polypropylene optic piercing technique for scleral fixation of a one-piece IOL can reposition subluxated or dislocated multifocal IOLs without adversely affecting the performance of the multifocal IOL or cause significant photic phenomena. In most cases in the literature, no postoperative complications occurred after flanged polypropylene scleral fixation [9, 13, 14, 29, 30], However, a case of postoperative endophthalmitis was reported recently [31]. Thus, flanged scleral fixation is a recently developed technique and will require long term follow up to monitor for potential future complications or IOL displacements [32, 33].

There are limitations to this study. First, there are only three cases enrolled in this study. Larger number of samples will be required to study the effectiveness of the proposed four-flanged polypropylene optic piercing technique for scleral fixation of one-piece multifocal IOLs. Second, MTF according to defocus was measured, but HOA was not measured in the experimental study. However, no photic phenomena such as starbursts or distortion were reported and postoperative internal total HOA was similar to postoperative corneal total HOA in all cases in this study. Third, the IOL used for surgery in the case series is different from the IOL used in the experimental study. Because the experimental study first confirmed that the new surgical method did not deteriorate the performance of the multifocal IOL, and then the surgical method was secondly applied to a patient who came to the hospital for dislocation of the multifocal IOL, the type of IOL could not be matched. However, the two multifocal IOLs differ in the material, optic design, and diffractive zone size (Table 1). The performance of the multifocal IOLs with a larger diffractive zone may be more affected by the optic piercing technique. In addition, as a softer optic material may contribute to optic distortion caused by 6 − 0 polypropylene sutures, optic piercing with two 6 − 0 polypropylene sutures may have a different effect on each IOL. Therefore, the optical bench test results of the TECNIS Synergy IOL cannot be applied to other multifocal IOLs, and additional experimental studies on other multifocal IOLs are needed.

## Conclusions

In conclusion, the four-flanged polypropylene optic piercing technique for one-piece multifocal IOL scleral fixation showed similar MTF at all defocus levels compared with the multifocal IOL without optic piercing and provided excellent clinical outcomes and IOL stability. Therefore, in cases of dislocated or subluxed one-piece multifocal IOL with C-loop haptics, scleral fixation of the dislocated IOL with four-flanged polypropylene optic piercing technique can be used to restore both distance and near/intermediate vision without the need to explant the IOL.

## Data Availability

The datasets during and/or analyzed during the current study available from the corresponding author on reasonable request.

## Abbreviations

IOLs: Intraocular lenses
MTF_RMS_: Root mean square of the modulation transfer function
UDVA: Uncorrected distance visual acuity
CDVA: Corrected distance visual acuity
UNVA: Uncorrected near visual acuity
CPD: Cycles per degree
CMOS: Complementary metal-oxide-semiconductor
MTF: Modulation transfer function
HOA: High-order aberration
SPSS: Statistical Package for Social Sciences

## References

[1] Keates RH, Pearce JL, Schneider RT (1987). Clinical results of the multifocal lens. J Cataract Refract Surg.

[2] Khandelwal SS, Jun JJ, Mak S, Booth MS, Shekelle PG (2019). Effectiveness of multifocal and monofocal intraocular lenses for cataract surgery and lens replacement: a systematic review and meta-analysis. Graefes Arch Clin Exp Ophthalmol.

[3] Kim JW, Eom Y, Park W, Song JS, Jeong JW, Park SK, Kim HM. Comparison of visual outcomes after two types of mix-and-match implanted trifocal extended-depth-of-focus and trifocal intraocular lenses. Graefes Arch Clin Exp Ophthalmol 2022. 10.1007/s00417-022-05710-w 35633381

[4] Kim JW, Eom Y, Chung HW, Song JS, Jeong JW, Park SK, Kim HM (2020). Factors for good near and distance visual outcomes of multifocal intraocular lens with inferior segmental near add. Graefes Arch Clin Exp Ophthalmol.

[5] Al-Shymali O, McAlinden C, Del Alio JL, Canto-Cerdan M, Alio JL (2022). Patients’ dissatisfaction with multifocal intraocular lenses managed by exchange with other multifocal lenses of different optical profiles. Eye Vis (Lond).

[6] Park SY, Eom Y, Lee YJ, Choi Y, Kim SJ, Song JS, Kim HM (2022). Scleral fixation of subluxated or dislocated multifocal and multifocal toric intraocular lenses. Graefes Arch Clin Exp Ophthalmol.

[7] Yamane S, Sato S, Maruyama-Inoue M, Kadonosono K (2017). Flanged intrascleral intraocular Lens fixation with double-needle technique. Ophthalmology.

[8] Canabrava S, Bernardino L, Batisteli T, Lopes G, Diniz-Filho A (2018). Double-flanged-haptic and capsular tension ring or segment for sutureless fixation in zonular instability. Int Ophthalmol.

[9] Canabrava S, Canedo Domingos Lima AC, Arancibia AEL, Bicalho Dornelas LF, Ribeiro G (2020). Novel double-flanged technique for managing Marfan syndrome and microspherophakia. J Cataract Refract Surg.

[10] Kelkar A, Kelkar J, Kothari A, Mehta H, Chitale S, Fogla R, Kelkar S (2018). Comparison of two modified Sutureless techniques of scleral fixation of intraocular Lens. Ophthalmic Surg Lasers Imaging Retina.

[11] Yuan A, Mustafi D, Banitt MR, Rezaei KA. Long-term outcomes of modified glued versus flanged intrascleral haptic fixation techniques for secondary intraocular lenses. Graefes Arch Clin Exp Ophthalmol 2022. 10.1007/s00417-022-05647-0 35389059

[12] Erakgun T. INTRAVITREAL NEEDLE TECHNIQUE for INTRASCLERAL HAPTIC FIXATION of POSTERIORLY DISLOCATED THREE-PIECE INTRAOCULAR LENSES. Retin Cases Brief Rep 2020. 10.1097/ICB.0000000000001102 PMC9750094 33323895

[13] Whang WJ, Kwon H, Jeon S (2020). Application of a four-flanged intrascleral fixation technique for toric and multifocal intraocular lenses. Am J Ophthalmol Case Rep.

[14] Canabrava S, Canêdo Domingos Lima AC, Ribeiro G (2020). Four-flanged intrascleral intraocular Lens fixation technique: no flaps, no knots, no glue. Cornea.

[15] Eom Y, Lee YJ, Park SY, Choi Y, Kim JW, Kim SJ, Song JS, Kim HM (2022). Cable tie technique for securing scleral fixation suture to intraocular lens. Am J Ophthalmol Case Rep.

[16] Rampat R, Gatinel D (2021). Multifocal and extended depth-of-focus intraocular lenses in 2020. Ophthalmology.

[17] Huh J, Eom Y, Yang SK, Choi Y, Kim HM, Song JS (2021). A comparison of clinical outcomes and optical performance between monofocal and new monofocal with enhanced intermediate function intraocular lenses: a case-control study. BMC Ophthalmol.

[18] Alba-Bueno F, Vega F, Millán MS. Design of a test bench for intraocular lens optical characterization. In: *Journal of physics: conference series*: 2011: IOP Publishing; 2011: 012105.

[19] Eom Y, Yang SK, Yoon EG, Choi JN, Ryu D, Kim DW, Kim JH, Song JS, Kim SW, Kim HM (2020). Multizonal Design Multifocal intraocular Lens-Induced Astigmatism according to Orientation. J Refract Surg.

[20] Kim JH, Eom Y, Park SY, Choi SY, Hwang HS, Kim JH, Song JS, Kim HM (2020). Rainbow halos occur less following implantation of extended range of vision one-piece intraocular lenses vs diffractive bifocal intraocular lenses. Int J Ophthalmol.

[21] Eom Y, Kim DW, Ryu D, Kim JH, Yang SK, Song JS, Kim SW, Kim HM (2017). Ring-shaped dysphotopsia associated with posterior chamber phakic implantable collamer lenses with a central hole. Acta Ophthalmol.

[22] Cole SC, Werner L, Schwiegerling J, Crandall A (2014). Visual aberrations in a multifocal intraocular lens with injection-related scratches. J Cataract Refract Surg.

[23] Smiddy WE, Sawusch MR, O’Brien TP, Scott DR, Huang SS (1990). Implantation of scleral-fixated posterior chamber intraocular lenses. J Cataract Refract Surg.

[24] Buckley EG (2007). Hanging by a thread: the long-term efficacy and safety of transscleral sutured intraocular lenses in children (an american Ophthalmological Society thesis). Trans Am Ophthalmol Soc.

[25] Price MO, Price FW, Werner L, Berlie C, Mamalis N (2005). Late dislocation of scleral-sutured posterior chamber intraocular lenses. J Cataract Refract Surg.

[26] Kim DW, Lee SC, Lee JH (2022). Scleral fixation of a hydrophobic acrylic intraocular Lens with Eyelets using 8 – 0 polypropylene suture. Korean J Ophthalmol.

[27] Bhojwani D, Vasavada AR, Vasavada V, Vasavada S, Praveen MR, Srivastava S (2020). Intraoperative performance and long-term postoperative outcomes after scleral fixation of IOLs with polytetrafluoroethylene suture. J Cataract Refract Surg.

[28] Domingues M, Brito P, Falcão M, Monteiro T, Falcão-Reis F (2011). Cupid fixation for repositioning subluxated intraocular lens. J Cataract Refract Surg.

[29] Assia EI, Wong JXH (2020). Adjustable 6 – 0 polypropylene flanged technique for scleral fixation, part 1: primary fixation IOLs in aphakia, capsular stabilizing devices, and aniridia implants. J Cataract Refract Surg.

[30] Belkin A, Yehezkeli V, Assia EI (2022). Trans-optic suture fixation of malpositioned intraocular lenses. Int Ophthalmol.

[31] Roditi E, Brosh K, Assayag E, Weill Y, Zadok D (2021). Endophtalmitis associated with flange exposure after a 4-flanged canabrava fixation techique. JCRS Online Case Reports.

[32] Canabrava S (2022). Comment on: Flange erosion/exposure and the risk for endophthalmitis. J Cataract Refract Surg.

[33] Canabrava SF, Rabelo NN, de Sousa Lima JL, de Nadai RF (2021). Exposed polypropylene flange in the Canabrava double-flanged polypropylene technique. JCRS Online Case Reports.

